# How Different Are Threshold and Other Specified Feeding and Eating Disorders? Comparing Severity and Treatment Outcome

**DOI:** 10.3389/fpsyg.2022.784512

**Published:** 2022-02-21

**Authors:** Samantha J. Withnell, Abbigail Kinnear, Philip Masson, Lindsay P. Bodell

**Affiliations:** Department of Psychology, University of Western Ontario, London, ON, Canada

**Keywords:** eating disorders, other specified feeding or eating disorder (OSFED), anorexia nervosa, bulimia nervosa, binge eating disorder, treatment outcome, impairment, DSM-5

## Abstract

**Background:**

Other Specified Feeding and Eating Disorders (OSFED) are characterized by less frequent symptoms or symptoms that do not meet full criteria for another eating disorder. Despite its high prevalence, limited research has examined differences in severity and treatment outcome among patients with OSFED compared to threshold EDs [Anorexia Nervosa (AN), Bulimia Nervosa (BN), and Binge Eating Disorder (BED)]. The purpose of the current study was to examine differences in clinical presentation and treatment outcome between a heterogenous group of patients with OSFED or threshold EDs.

**Method:**

Patients with threshold EDs (*AN* = 42, *BN* = 50, BED = 14) or OSFED (*n* = 66) presenting for eating disorder treatment completed self-report questionnaires at intake and discharge to assess eating disorder symptoms, depression symptoms, impairment, and self-esteem.

**Results:**

At intake, OSFED patients showed lower eating concerns compared to patients with BN, but similar levels compared to AN and BED. The OSFED group showed higher restraint symptoms compared to BED, and similar restraint to AN and BN. Global symptoms as well as shape and weight concerns were similar between OSFED and threshold ED groups. There were no differences between diagnostic groups in self-esteem, depression scores, or symptom change from intake to discharge.

**Discussion:**

Our findings suggest that individuals with OSFED showed largely similar ED psychopathology and similar decreases in symptoms across treatment as individuals diagnosed with threshold EDs. Taken together, findings challenge the idea that OSFED is less severe and more resistant to treatment than threshold EDs.

## Introduction

There are three named eating disorders (EDs) in the current version of the Diagnostic and Statistical Manual of Mental Disorders (DSM-5; American Psychiatric Association, 2013), including anorexia nervosa (AN), bulimia nervosa (BN), and binge eating disorder (BED). However, the DSM-5 also has a fourth category for EDs labeled “Other Specified Feeding and Eating Disorders” (OSFED) that represents clinically significant eating pathology that does not meet full criteria for AN, BN, or BED. Examples of OSFED cases provided by the DSM-5 include those with less frequent symptoms (e.g., subthreshold BN/BED) or missing certain symptoms (e.g., atypical AN, purging disorder). Although OSFED is often viewed as a “residual” diagnosis, the category consistently represents the largest proportion of ED diagnoses (Eddy et al., 2008; Stice et al., 2009; Mustelin et al., 2016; Vo et al., 2017; Mitchison et al., 2020). Despite its high prevalence, OSFED has received much less research attention compared to threshold EDs (i.e., AN, BN, and BED) since its introduction in DSM-5.

The OSFED category includes heterogenous symptom presentations, which has led to concerns that the diagnostic label may not convey useful information about illness course, prognosis, or treatment needs (Le Grange et al., 2012). Additionally, because the label is often used to capture “subthreshold” cases, OSFED diagnoses may be perceived as less severe. Although individuals with OSFED may present with symptoms of lower frequency or intensity than threshold EDs, by definition, they also experience high functional impairment and distress (American Psychiatric Association, 2013). OSFED patients also experience high prevalence of psychiatric comorbidities, non-suicidal self-injury (NSSI), and suicidal ideation and attempts (Schmidt et al., 2008; Le Grange et al., 2012; Mustelin et al., 2016; Riesco et al., 2018). Further, previous studies have found few differences between OSFED and threshold ED groups in terms of eating pathology severity as well as cognitive, genetic, and environmental risk factors (Thomas et al., 2009; Fairweather-Schmidt and Wade, 2014; Krug et al., 2021). The few existing studies directly comparing treatment outcomes between patients with threshold EDs and OSFED presentations are inconsistent, with one finding similar treatment outcomes (Schmidt et al., 2008) and another finding poorer outcomes and higher rates of dropout among OSFED patients (Fernández-Aranda et al., 2021). Further, only one study to date has examined outcomes among OSFED patients receiving outpatient enhanced CBT (CBT-E), finding low motivation to change as well as high rates of dropout in this group (Riesco et al., 2018).

An important limitation of previous literature is that diagnosis of OSFED and inclusion of subcategories have been defined differently across studies. In prior editions of the DSM, the diagnostic category [formerly referred to as Eating Disorder Not Otherwise Specified (EDNOS)] encapsulated all “residual” cases in which symptoms did not meet full criteria for AN or BN. Thus, various symptom presentations were included within the same diagnosis (e.g., subthreshold AN and BED). Indeed, in the DSM-5, BED was moved out of the EDNOS category and became a threshold ED along with AN and BN. Studies conducted before the introduction of DSM-5 inconsistently included patients with BED within the EDNOS group (Thomas et al., 2009; Le Grange et al., 2012). Other studies have arbitrarily excluded certain categories, such as BED of low frequency or duration, or used eligibility criteria no longer included in DSM-5, such as requiring low weight and amenorrhea for diagnoses of subthreshold and threshold AN (Stice et al., 2009; Fairweather-Schmidt and Wade, 2014; Riesco et al., 2018; Krug et al., 2021). Further, studies comparing treatment outcomes have included patient groups receiving different treatment modalities (i.e., family-based vs. individual CBT). Inclusion or exclusion of specific symptom presentations or treatment modalities may contribute to inconsistent findings of severity and outcome among OSFED patients compared to those with threshold EDs in existing research.

The purpose of the current study was to extend the current literature by describing differences in clinical presentation and treatment outcome between a group of OSFED patients and those with threshold EDs. Analyses were not preregistered; thus, this study should be considered exploratory. This study contributes to the current literature on OSFED by including patients using DSM-5 diagnostic criteria as well as comparing differences at intake and discharge from treatment between diagnostic groups.

## Method

### Participants and Procedure

Individuals presenting to a community-based eating disorder treatment service consented to completing program evaluation questionnaires (*N* = 296). The vast majority of patients presenting to this service identified as White and female. Data collection was approved by the hospital’s Research Ethics Board. Of those who consented, 172 completed intake questionnaires and were able to be included in the current study. This sample included 106 patients diagnosed with a threshold ED (*AN* = 42, *BN* = 50, BED = 14) and 66 diagnosed with OSFED. OSFED cases included those with atypical AN (*n* = 6), subthreshold/low frequency BN (*n* = 2), subthreshold/low frequency BED (*n* = 1), purging disorder (*n* = 2), or other (e.g., food restriction and shape/weight concerns) (*n* = 57). ED diagnoses were determined by clinician-rated interviews using a modified version of the Eating Disorder Examination (Fairburn et al., 2008). All interviews were conducted by one assessor and reviewed by the third author to confirm diagnosis. Treatment generally consisted of manualized CBT-E across a range of settings (outpatient, day treatment, and brief residential; Fairburn et al., 2003). Participants completed self-report questionnaires again at discharge from treatment (*N* = 97). Listwise deletion was used for mixed ANOVA analyses for participants missing data at treatment discharge, resulting in 95 participants included in these analyses. There were no differences between ED diagnostic groups in the proportion who were missing data at discharge (χ^2^ = 6.91, *p* = 0.075).

### Measures

#### Beck Depression Inventory

The Beck Depression Inventory (BDI-II; Beck et al., 1996) is a clinical tool assessing severity of depressive symptoms. It consists of 21 items rated on a Likert scale from 0 to 3. Total scores are derived from the sum of all items, with higher scores representing more severe depressive symptoms. The BDI-II has shown good reliability and validity in clinical and non-clinical samples (Erford et al., 2016), and showed excellent reliability in the current sample at intake (α = 0.93).

#### Rosenberg Self-Esteem Scale

The Rosenberg Self-Esteem Scale (RSES; Rosenberg, 1965) was used to measure participants’ global self-esteem. This measure contains 10 items on a 4-point Likert-type scale from “Strongly Agree” to “Strongly Disagree.” Item content contains both positive and negative feelings about the self. The RSES has demonstrated good reliability in large samples (Sinclair et al., 2010), and showed good reliability in the current sample at intake (α = 0.87).

#### Eating Disorder Examination Questionnaire

Eating disorder symptoms were measured using the Eating Disorder Examination Questionnaire (EDE-Q; Fairburn and Beglin, 2008). The EDE-Q is a 22-item measure that assesses eating pathology over the past 28 days. Symptoms are rated on a seven-point Likert scale from 0 (“no days”) to 6 (“every day”), with higher scores representing greater symptomology. Items can be scored together as an indicator of global eating pathology. The EDE-Q also can be divided into four subscales: Restraint, Eating Concerns, Shape Concerns, and Weight Concerns. The EDE-Q has demonstrated good reliability and validity in clinical samples (Berg et al., 2012). At intake, reliability in the current sample was 0.82 for global scores and ranged from 0.71 (eating concerns) to 0.87 (shape concerns) for subscale scores.

#### Clinical Impairment Assessment Questionnaire

Severity of psychosocial impairment from ED symptoms was measured using the Clinical Impairment Assessment questionnaire (CIA; Bohn and Fairburn, 2008). The CIA contains 16 items that assess impairment in mood and self-perception, cognitive functioning, interpersonal functioning, and work performance over the past 28 days. Items are rated on a 4-point Likert type scale, from 0 (“Not at all”) to 3 (“A lot”), with higher scores representing greater impairment. Internal consistency of the CIA at intake was 0.93.

### Data Analysis

Analyses were conducted in SPSS 27 (IBM Corp, 2020). One-way analysis of variance (ANOVA) was used to examine whether diagnostic groups differed at intake on ED symptoms, impairment, depression, and self-esteem scores. To examine whether diagnostic groups differed on change in ED symptoms and other psychopathology from intake to treatment discharge, 2 (treatment: intake, discharge) × 4 (diagnosis: AN, BN, BED, and OSFED) mixed between-within subjects ANOVAs were conducted. Given the large number of analyses, a familywise alpha level of *p* < 0.01 was used to determine significance. Pairwise comparisons with Bonferroni correction were used to follow-up any significant main effects or interactions. Bonferroni-adjusted *p*-values are reported for pairwise comparisons. Cohen’s d effect sizes were calculated to describe the strength of the finding. The following guidelines were used for interpretation: 0.20 (small), 0.50 (medium), >0.80 (large).

## Results

### Differences at Intake

Descriptive and symptom characteristics of diagnostic groups at treatment intake are listed in Table 1. There were significant differences between diagnostic groups in age, with *post-hoc* comparisons indicating that OSFED patients were younger on average compared to those with BED (*p* < 0.001). However, OSFED patients were similar in age to patients with AN and BN, who were also significantly younger than patients with BED. There were also significant differences between all four groups on BMI at intake. On average, BMI among the OSFED group was higher compared to the AN group, but lower compared to BN and BED. There were no differences between diagnostic groups on age of ED onset.

**TABLE 1:** Descriptive characteristics and symptoms across diagnostic group at treatment intake.

		AN		BN		BED		OSFED				
	*N*	*M*	*SD*	*M*	*SD*	*M*	*SD*	*M*	*SD*	*F*	*p*	η ^2^
Age	292	28.70^a^	10.82	30.73^b^	11.88	39.95^a,b,c^	12.71	29.26^c^	11.90	**10.05**	** < 0.001**	0.094
BMI	282	17.91^a^	2.36	29.66^a^	22.38	38.75^a^	10.55	23.46^a^	6.18	**27.22**	** < 0.001**	0.225
Age at first ED	291	18.58	7.01	16.85	5.36	21.67	12.07	19.08	7.55	3.67	0.013	0.036
EDE-Q global	172	4.45^b^	0.16	4.60^a^	0.15	3.42^a,b^	0.28	4.09^a^	0.13	**5.17**	**0.002**	0.085
EDE-Q restraint	172	3.84^a^	0.24	3.90^b^	0.22	2.20^a,b,c^	0.41	3.57^c^	0.19	**4.85**	**0.003**	0.080
EDE-Q eating	172	3.78	0.20	4.20^a^	0.19	3.19^a^	0.35	3.27^a^	0.16	**5.56**	**0.001**	0.090
EDE-Q shape	172	5.35	0.16	5.32	0.15	4.80	0.28	5.02	0.13	1.83	0.143	0.032
EDE-Q weight	172	4.82	0.19	4.96	0.17	3.89	0.33	4.53	0.15	3.38	0.020	0.057
CIA	171	39.05	1.46	38.61	1.32	32.21	2.50	35.29	1.15	3.11	0.028	0.053
BDI	170	19.78	1.98	24.53	1.77	25.83	3.35	21.18	1.54	1.61	0.188	0.028
RSES	171	9.78	0.87	9.60	0.79	12.33	1.48	10.51	0.68	1.03	0.380	0.018

*Bolded text indicates test significant at p < 0.01. Superscripts indicate significant post-hoc comparisons. AN, Anorexia Nervosa; BN, Bulimia Nervosa; BED, Binge Eating Disorder; OSFED, Other Specified Feeding or Eating Disorder; BMI, Body Mass Index; EDE-Q, Eating Disorder Examination Questionnaire; CIA, Clinical Impairment Assessment; BDI, Beck Depression Inventory; RSES, Rosenberg Self-Esteem Scale.*

Diagnostic groups did not differ on depression symptoms or self-esteem at intake. There were significant differences between diagnostic groups on global EDE-Q scores at intake; however, there were no differences between patients with OSFED compared to those with AN (*p* = 0.526), BN (*p* = 0.063), or BED (*p* = 0.354). The groups also differed significantly on restraint scores, with participants with BED exhibiting significantly lower restraint scores compared to all other groups, including OSFED (*p* = 0.017). Significant differences also were found for eating concerns, with OSFED patients showing lower eating concerns compared to those with BN (*p* < 0.001, *d* = 0.71) but similar concerns as patients with AN and BED (*p* = 0.310, *d* = 0.39 and *p* = 1.000, *d* = 0.06, respectively). There were no differences in weight concerns, shape concerns or impairment between the OSFED group and any of the threshold EDs.

### Treatment Outcomes

As expected, there was a significant main effect of treatment on EDE-Q global scores, indicating that ED symptoms overall decreased from intake to discharge across diagnosis (see Table 2). However, there was no main effect of diagnosis or interaction between treatment and diagnosis, indicating that change in global symptoms on the EDE-Q over treatment did not differ between diagnostic groups. These results were consistent for restraint, weight, shape, and eating concerns such that there was a significant main effect of treatment, but no main effect of diagnosis and no significant interaction between treatment and diagnosis on any of these outcomes. As with the findings at intake, patients with BED showed significantly lower restraint scores over treatment compared to all other groups. These findings suggest that all EDE-Q subscale scores decreased from intake to discharge similarly across diagnostic groups.

**TABLE 2:** Tests of main effects and interaction of treatment (intake, discharge) and diagnosis on ED symptoms.

	EDE-Q global			EDE-Q restraint			EDE-Q eating			EDE-Q shape			EDE-Q weight		
	*F*	*p*	η _*p*_^2^	*F*	*p*	η _*p*_^2^	*F*	*p*	η _*p*_^2^	*F*	*p*	η _*p*_^2^	*F*	*p*	η _*p*_^2^
Treatment	**126.28**	**<0.001**	0.58	**82.26**	**<0.001**	0.47	**97.24**	**<0.001**	0.52	**77.87**	**<0.001**	0.46	**72.57**	**<0.001**	0.44
Diagnosis	2.53	0.062	0.07	3.14	0.029	0.09	1.17	0.327	0.04	1.57	0.202	0.05	2.18	0.096	0.07
Treatment × Diagnosis	0.73	0.538	0.02	1.68	0.176	0.05	0.72	0.543	0.02	1.01	0.391	0.03	0.61	0.611	0.01

*Bolded text indicates test significant at p < 0.01. EDE-Q, Eating Disorder Examination Questionnaire.*

Findings regarding the impact of diagnosis on clinical impairment, depression, and self-esteem over treatment also are consistent with findings on ED symptoms. Specifically, there was a significant main effect of treatment on clinical impairment, indicating that impairment significantly decreased from intake to discharge across groups, *F*(1, 90) = 89.93, *p* < 0.001. However, the interaction between treatment and diagnosis on clinical impairment was not significant, *F*(3, 90) = 0.15, *p* = 0.929. Moreover, there was a main effect of treatment on depressive symptoms, *F*(1, 90) = 25.79, *p* < 0.001, but no main effect of diagnosis, *F*(3, 90) = 0.24, *p* = 0.868, and no significant interaction between treatment and diagnosis on depressive symptoms, *F*(3, 90) = 1.02, *p* = 0.386. Finally, there was a main effect of treatment *F*(1, 90) = 41.50, *p* < 0.001, but no main effect of diagnosis *F*(3, 90) = 1.34, *p* = 0.266 or interaction between treatment and diagnosis on self-esteem *F*(3, 90) = 0.48, *p* = 0.699.

## Discussion

The purpose of the current study was to examine differences between patients diagnosed with OSFED compared to threshold EDs on eating and other psychopathology and response to treatment. Our findings indicated that ED psychopathology among OSFED patients was largely similar to patients with threshold EDs, with a few exceptions. Namely, eating concerns were lower among OSFED patients compared to BN patients, but similar to AN and BED. Restraint symptoms were also higher in OSFED patients compared to BED. There were no differences between OSFED and threshold ED groups on weight concerns, shape concerns, impairment, depressive symptoms, or self-esteem at intake. Further, there were no interactions between diagnosis and treatment on psychopathology measures, indicating that all diagnostic groups showed similar decreases in general psychopathology and ED symptoms and impairment from intake to discharge. Taken together, our findings suggest that individuals presenting to treatment with OSFED diagnoses experience similar levels of psychopathology compared to individuals with threshold EDs and show similar benefits from a transdiagnostic CBT based treatment. These findings provide further support for the transdiagnostic model of eating disorders, in which the shared underlying pathology of overvaluation of weight and shape may drive ED behaviors (Fairburn et al., 2003).

Our findings of greater eating concerns on the EDE-Q among BN patients compared to OSFED are consistent with previous research identifying greater eating pathology in BN (Eddy et al., 2008; Thomas et al., 2009). However, there were no differences in overall EDE-Q scores, restraint, shape concerns, or weight concerns between OSFED and BN. Previous studies have similarly found no differences in cognitive symptoms or treatment outcomes between BN and OSFED patients with subthreshold binge episodes, suggesting that despite differences in behavior frequency, differentiating between these two groups may not be clinically useful (Schmidt et al., 2008). The frequency of binge eating or compensatory behaviors that best differentiates BN from less severe or “subthreshold” forms of BN may also be lower than cut-offs currently included in DSM-5 (Johnson et al., 2021). Thus, more research is needed to examine whether binge-episode criteria should be further relaxed for BN diagnoses.

Symptoms at intake were largely similar between OSFED, AN, and BED, with the exception that the OSFED group showed greater restraint compared to the BED group. These findings contrast somewhat with previous studies finding no differences between these groups (Stice et al., 2009; Thomas et al., 2009). Higher restraint in the OSFED group in our sample may reflect low representation of clinical presentations characterized by uncontrolled eating (i.e., BN/BED of low frequency or duration; Wilfley et al., 2000). However, patients with BED also showed lower overall severity, weight and eating concerns, and clinical impairment compared to patients with AN and BN. Thus, in this treatment-seeking community based transdiagnostic sample, OSFED appeared to be more similar to AN and BN compared to BED. Indeed, the shift of BED diagnosis from the EDNOS category in DSM-IV to a distinct ED in DSM-5, despite the lower severity of symptoms found in the BED group in the current study, challenges the utility of current distinctions between OSFED and “threshold” EDs based on assumptions of severity.

Our findings also have important implications for treatment as patients with OSFED showed similar positive responses to treatment as those with threshold EDs. Indeed, general and ED psychopathology improved over the course of treatment across all diagnostic groups. Thus, the current study may lend support to transdiagnostic treatment models including CBT-E for individuals with OSFED diagnoses. These findings are consistent with Schmidt et al. (2008), but contrast with other studies finding poorer treatment response among OSFED patients (Riesco et al., 2018; Fernández-Aranda et al., 2021). Inconsistencies in previous literature may be due to different clinical presentations included or excluded in the OSFED group. For example, subcategories within OSFED were not reported by Fernández-Aranda et al. (2021), and other studies excluded patients reporting binge eating in the absence of compensatory behaviors (i.e., BED of low frequency or duration). Differences in treatment setting and modality may also contribute to clinical outcomes, as previous studies compared patients receiving guided self-help, outpatient, and residential treatments. Although findings from the current study suggest that individuals with “residual” symptom presentations benefit from existing ED treatments, researchers should further investigate whether different treatments may differentially impact outcomes across individuals with OSFED and threshold EDs.

Taken together, the limited differences in symptom severity and response to treatment among patients diagnosed with OSFED or threshold EDs in the current study may challenge clinician assumptions that an OSFED diagnosis reflects lower severity or clinical significance. The OSFED diagnosis includes example presentations reflecting “subthreshold” symptoms (i.e., BN/BED of low frequency or duration, atypical AN) as well as presentations considered nosologically distinct (i.e., purging disorder, night eating syndrome). Yet, our findings that OSFED patients showed similar severity and treatment outcomes compared to threshold EDs, suggests that distinctions based on behavioral frequencies may not be clinically meaningful. Indeed, Wade and O’Shea (2015) similarly found severity and impairment levels comparable to threshold EDs among a sample of adolescents presenting with food restriction, excessive exercise, and high weight/shape concerns who did not meet criteria for threshold EDs or OSFED (i.e., were characterized as Unspecified Feeding or Eating Disorder). Thus, cut-offs based on behavioral frequencies or body weight may not provide reliable assessment of severity. Instead, cognitive features including drive for thinness have been explored as more useful markers of severity (Davenport et al., 2015; Krug et al., 2021). However, more research attention is needed to examine characteristics, prognosis, and treatment needs of this unique population not presently well captured by current diagnostic systems.

The current study improves upon previous research comparing OSFED to threshold EDs by investigating outcomes across treatment among a large sample receiving CBT based treatment. Our patient data came from a naturalistic treatment setting, which may benefit the generalizability of our findings to other CBT-based settings. However, this design also led to high levels of missing data in our sample and may limit the generalizability of our findings to more diverse samples. Although we included heterogenous OSFED presentations which addressed inconsistencies in previous research, our findings may be limited by the small number of OSFED patients presenting with “subthreshold” BN and BED presentations, which prevented direct comparisons between “threshold” and “subthreshold” presentations within diagnoses. These presentations may reflect earlier stages of illness that are less likely to be captured in treatment-seeking samples (Eddy et al., 2008; Schmidt et al., 2008; Stice et al., 2009). Selection bias also may have influenced the makeup of our sample, such that individuals experiencing less severe forms of OSFED may not have sought treatment. OSFED may also be more prevalent in populations who are less likely to present for or receive treatment, such as men and ethnic minorities (Eddy et al., 2008; Thomas et al., 2009; Mitchison et al., 2020). Future research would benefit from replication and extension of our findings to clinical populations in other treatment settings and modalities. Additionally, future studies employing equivalence tests or Bayesian analyses could be used to confirm similarities between OSFED and other ED diagnoses. Further investigation is also needed examining whether OSFED presentations should be included as distinct named EDs in future iterations of the DSM.

## Data Availability Statement

The datasets presented in this article are not readily available however completely anonymized data may be available upon reasonable request. Requests to access the datasets should be directed to the corresponding author.

## Ethics Statement

The study was reviewed and approved by the Western Health Sciences Research Ethics Board Lawson Health Research Institute Western University London, ON, Canada. The patients/participants provided their written informed consent to participate in this study.

## Author Contributions

SW conducted data analyses and wrote the first draft of the manuscript. AK provided feedback and revisions to the manuscript. PM designed and oversaw the data collection and provided feedback to the manuscript. LB conceptualized the study, assisted with data analyses, and provided feedback and revisions to the manuscript. All authors contributed substantially to the conceptualization of the study and writing of the manuscript.

## Conflict of Interest

The authors declare that the research was conducted in the absence of any commercial or financial relationships that could be construed as a potential conflict of interest.

## Publisher’s Note

All claims expressed in this article are solely those of the authors and do not necessarily represent those of their affiliated organizations, or those of the publisher, the editors and the reviewers. Any product that may be evaluated in this article, or claim that may be made by its manufacturer, is not guaranteed or endorsed by the publisher.
